# Nursing Interns’ Disgust Sensitivity, Ageism and Gerontological Nursing Career Motivation: A Network Analysis

**DOI:** 10.1155/jonm/9944227

**Published:** 2026-05-18

**Authors:** Yiqian Tang, Chenxi Zhu, Liling Xu, Lilu Wang, Yawen Hu, Lianlian Zhu, Yechun Gu, Hongbo Xu

**Affiliations:** ^1^ School of Nursing, Wenzhou Medical University, Wenzhou, China, wmu.edu.cn; ^2^ General Surgery Department, Wenzhou Hospital of Integrated Traditional Chinese and Western Medicine, Wenzhou, China, wzszxy.com

**Keywords:** ageism, disgust sensitivity, gerontological nursing career motivation, network analysis, nursing interns

## Abstract

**Background:**

Disgust sensitivity and ageism significantly affect nursing interns’ motivation to work in gerontological nursing. However, the complex interactions between these factors remain underexplored.

**Objective:**

This study used network analysis to explore relationships among nursing interns’ disgust sensitivity, ageism and motivation to pursue a career in gerontological nursing and to identify key nodes in this network.

**Methods:**

This study recruited 698 nursing interns. The Disgust Scale–Revised (DS‐R), the Fraboni Scale of Ageism and the Gerontological Nursing Career Motivation Questionnaire were used as measures. Centrality and bridge centrality indices were used to identify central and bridge symptoms. Network stability was assessed using a case‐dropping bootstrap procedure. The network comparison test was used to analyse differences in networks by gender and residence. Bayesian networks were used to investigate possible probabilistic connections among variables.

**Results:**

Network analysis identified ‘antilocution’ (D5) as the most central node in the overall network, whereas ‘expectation’ (D7) had the highest bridge strength. Bayesian network analysis further revealed that ‘avoidance’ (D4) had the greatest probabilistic influence and was strongly linked to other nodes associated with ageism and disgust sensitivity. ‘Practicality’ (D9) and ‘importance’ (D10) also showed significant probabilistic associations within the motivation domain. The network structure proved stable and reliable, with no significant differences attributable to gender or to residence.

**Conclusions:**

This study highlights the complex relationships among disgust sensitivity, ageism and motivation for a career in gerontological nursing. It identifies ‘antilocution’ (D5) in ageism and ‘expectation’ (D7) in career motivation as central nodes, associated with negative attitudes and lower career identity. Additionally, ‘avoidance’ (D4) behaviour shows a probabilistic association with higher levels of disgust sensitivity and with ageism. These findings suggest targeted interventions to improve the well‐being and career motivation of nursing interns in gerontological nursing.

## 1. Introduction

The shortage of geriatric nursing staff has consistently been a major challenge in public health. As the global population ages, this problem is becoming more critical than ever before [1, 2]. As demand for high‐quality elderly care services grows, it has become increasingly important to recruit and retain highly qualified nursing professionals. Nursing interns represent the future gerontological nursing workforce, playing a crucial role in addressing this gap. However, their willingness to pursue this kind of work is generally low [3, 4]. This lack of motivation among nursing interns not only worsens workforce shortages but may also affect the overall quality of care for older adults. Notably, current research often focuses on the linear effects of a single variable (such as work experience or work environment) [5, 6], while neglecting the complex interconnections between psychological and social factors. Taking such a narrow approach might overlook important underlying factors that affect career choice. Therefore, an in‐depth exploration of the interplay among multiple factors is important for increasing nursing interns’ willingness to pursue a career in geriatric nursing.

This study integrates the structures of three variables, namely disgust sensitivity, ageism and career motivation, into a unified analytical framework. By constructing partial correlation networks and Bayesian network models, it identifies key nodes and probabilistic association patterns, thereby providing valuable theoretical support for nursing education and targeted interventions. Ultimately, these findings could inform the development of educational programmes and policy initiatives that promote more positive attitudes toward geriatric nursing among future healthcare professionals.

## 2. Background

The global population is ageing more quickly than ever before. United Nations data show that in 2021, there were 761 million people aged 65 and over—a number expected to reach 1.6 billion by 2050 [7]. The United Nations World Population Prospects 2022 Population Projection Program indicated that by 2050, China’s population aged 60 years and over will exceed 500 million, accounting for 38.81% of the total population [8], thus entering a stage of severe population ageing. The growing elderly population has steadily widened the gap between the supply of and the demand for elderly care professionals [9]. Nursing interns are a core talent reserve, and their career choice directly affects the industry’s sustainable development. However, previous studies have shown that nursing interns are less inclined to pursue a career in geriatric care. For example, a study from Israel found that 61% of soon‐to‐be‐employed nursing interns said they did not intend to work in geriatric care, and only 12% considered it a promising field [10]. In China, Wang et al.’s study found that 50.27% of Chinese undergraduate nursing students were willing to work in gerontological nursing [11].

Furthermore, a variety of factors influence nursing interns’ career choices, and understanding a specific psychological trait is crucial to identifying the key factor underlying nursing students’ lack of interest in geriatric nursing. Disgust, one of the basic emotions shared by humans, is a multicomponent emotion, encompassing affective, cognitive and physical aspects, and it can be triggered by multiple stimuli [12]. It is always triggered when the organism is exposed to a pathogen or a health threat [13]. Disgust sensitivity is the propensity to experience disgust, with individual differences in sensitivity arising from factors such as learning history and genetic variation [14]. Prior research has shown that disgust is an important influence on career choice [12, 15, 16]. Moreover, disgust affects an individual’s thoughts and behaviours [17]. Compared with other specialities, geriatric nursing encounters more stimuli that can provoke disgust, such as physiological phenomena such as pressure sores, incontinence or body odour in the elderly, as well as complex moral and ethical issues that may arise at the end‐of‐life care [18]. As a result, nursing interns may develop heightened disgust sensitivity in clinical settings, which could deter them from pursuing a career in geriatric nursing.

Ageism refers to stereotypes, biases and discriminatory behaviours toward older adults because of their age [19]. It consists of two main aspects: discriminatory attitudes, including inherent prejudices against older persons, such as the perception that they are lonely or lack value; and discriminatory behaviours, such as avoiding older persons or ignoring their needs [20]. Previous research has found that the greater the nursing intern’s prejudice against older adults, the lower their motivation to engage in geriatric care [3, 21]. One study has already found that nursing interns who hold negative perceptions and attitudes toward older adults may develop values that steer them away from the geriatric nursing profession [22].

Previous research has emphasised the importance of nursing interns’ disgust sensitivity and ageism in motivating careers in gerontological nursing; however, there has been no systematic investigation of their relationship. Conventional approaches such as correlation and regression analysis may fall short of revealing the intricate interactions and network dynamics among them [23]. Furthermore, using cross‐sectional data, it is not possible to determine the causal direction among symptoms. These limitations constrain the understanding of the core connections among disgust sensitivity, ageism and motivation to pursue a career in gerontological nursing.

In contrast, network analysis offers notable benefits for examining these complex relationships. By modelling these dimensions as interconnected nodes in a network, network analysis reveals complex dynamics that traditional methods cannot capture. This approach can identify key nodes or central symptoms within a network, offering insights into the complex relationships among these variables [24–27]. This method also includes the concepts of central symptoms and bridging nodes, allowing researchers to identify central symptoms that strongly influence the network, as well as bridging symptoms that connect different symptom clusters [28–30]. Using network analysis helps to explore how variables interact and assist in optimising interventions to boost nursing interns’ motivation to pursue a career in geriatric care. Furthermore, using directed acyclic graphs (DAGs) and Bayesian network methods, probabilistic directional associations can be explored even in cross‐sectional data [31]. These methods complement each other and offer new perspectives on their interrelationships. Therefore, employing network analysis to examine the intrinsic linkages among these three variables offers a novel approach to understanding their complex relationships and identifying the most critical targets for enhancing intervention effectiveness.

## 3. The Study

### 3.1. Aim

This study aimed to examine the relationships among disgust sensitivity, ageism and motivation to pursue a career in gerontological nursing among nursing interns using a network analysis approach, thereby providing a theoretical foundation for developing targeted interventions to boost career motivation in geriatric care.

## 4. Materials and Methods

### 4.1. Study Design and Participants

Between January and June 2024, this cross‐sectional study recruited nursing interns from 12 large, urban, tertiary‐level general hospitals in Zhejiang Province, China, using a continuous sampling method. The inclusion criteria for this study were as follows: (1) fourth‐year college nursing interns who have completed at least 8 months of clinical practice and (2) voluntary participation in the study. Participants with a history of mental illness, past or current, were excluded.

According to the criteria proposed by Kendall [32], the sample size should be 10–20 times the number of entries. The present study used a sample estimate of 20 times. This study included 10 items on general sociodemographic characteristics, 3 dimensions of the Chinese version of the Disgust Scale–Revised (DS‐R), 3 dimensions of the Fraboni Scale of Ageism and 2 dimensions of the Gerontological Nursing Career Motivation Questionnaire. The sample size was calculated as 360. Accounting for 20% invalid questionnaires, the target sample size was 450.

### 4.2. Data Collection

The researchers contacted the nursing departments at the hospitals to explain the purpose of the research and obtained consent before distributing the online questionnaires to the nursing interns. Before completing the questionnaire, participants were informed of the survey’s purpose and significance, as well as the instructions and key points for completing it. The questionnaires were collected online by trained surveyors using the Questionnaire Star platform (http://www.wjx.cn), which is widely used in China and operates through WeChat, the most popular communication application [33]. It took about 20 min to finish.

A total of 746 nursing interns participated in this study, and 698 questionnaires were deemed valid (excluding those with patterned responses, incomplete answers, duplicates or very short completion times), yielding a valid response rate of 93.6%.

### 4.3. Data Measures

The study used a self‐designed questionnaire alongside three standardised questionnaires. The self‐designed questionnaire was used to measure participants’ general sociodemographic characteristics. The standardised questionnaires were used to measure disgust sensitivity, ageism and motivation for a career in gerontological nursing, demonstrating good reliability and validity, and have been widely adopted in China.

#### 4.3.1. Sociodemographic Questionnaire

Based on previous research and expert consultation, a general information questionnaire was designed to include gender, residence, one‐child status, self‐reported family financial status, primary caregiver, whether living with elderly relatives, first‐choice major in nursing, geriatric care experience and geriatric nursing course learning experience.

#### 4.3.2. Disgust Sensitivity

This study used the Chinese version of the DS‐R, developed by Olatunji et al. [34] and translated and revised by Li [35], to assess the disgust sensitivity of nursing interns. The scale comprises 27 items, including 2 polygraph items that are not scored. It comprises three dimensions: core disgust (12 items), animal‐reminder disgust (8 items) and contamination‐based disgust (5 items). A 5‐point Likert scale was used for scoring, with items 1, 6 and 10 reverse‐coded. Higher scores on each dimension indicate the level of disgust sensitivity among nursing interns in that area. The higher the total score, the greater the overall disgust sensitivity among the nursing interns. In this study, Cronbach’s *α* for the scale was 0.86.

#### 4.3.3. Ageism

The Chinese version of the Fraboni Scale of Ageism was used to assess the extent of ageism among nursing interns. The scale was originally developed by Fraboni et al. [36], and the Chinese version was translated and revised by Fan et al. [37]. It comprises 22 items across three dimensions: avoidance, antilocution and discrimination. A 4‐point Likert scale was used (1 = *strongly disagree*, 4 = *strongly agree*). Higher scores on each dimension indicate stronger ageist attitudes in that area. The scale’s Cronbach’s *α* in this study is 0.88.

#### 4.3.4. Gerontological Nursing Career Motivation

Nursing interns’ intention to pursue careers in geriatric nursing was assessed using the Gerontological Nursing Career Motivation Questionnaire. The questionnaire was developed based on the expectation–value theory by Cheng [38]. It consists of 20 items in two parts: the expectation questionnaire (6 items) and the value questionnaire (14 items). The value questionnaire was further divided into four dimensions: interest (3 items), practicality (3 items), importance (5 items) and cost (3 items). Both questionnaires are scored on a 5‐point Likert scale, ranging from 1 (*strongly disagree*) to 5 (*strongly agree*). The higher the total score, the greater the nursing students’ value placed on and expectations of geriatric nursing. In this study, Cronbach’s *α* for the questionnaire was 0.95.

### 4.4. Data Analysis

All statistical analyses in this study were conducted using SPSS (Version 27.0) and *R* (Version 4.4.2). A series of network analyses, including network estimation, stability assessment and comparison, was conducted. Additionally, the mean, standard deviation (SD), skewness and kurtosis for each dimension were computed.

#### 4.4.1. Network Estimation

The network in this study comprises 11 dimensions: 3 from the DS‐R and the Ageism Scale, the expectation dimension from the Gerontological Nursing Career Motivation Questionnaire and 4 subdimensions of the value dimension (interest, practicality, importance and cost). Each dimension was treated as a ‘node’, and the connections between them were ‘edges’. The colour of each edge indicated the direction of the correlation: green for positive correlations and red for negative ones. Additionally, the thickness of the edges in the network diagram indicates the strength of the correlation between connected nodes. Thinner edges indicate weaker correlations between nodes; conversely, the stronger [28]. This study utilised the extended Bayesian information criterion (EBIC) and the least absolute shrinkage and selection operator (LASSO) network models (*γ* = 0.5) [28, 39] to construct a highly interpretable network linking disgust sensitivity, ageism and gerontological nursing career motivation. The *R* packages qgraph (Version 1.9.8) and bootnet (Version 1.6) were used for network estimation and visualisation [28, 40, 41].

Network centrality indices include strength, betweenness and closeness, but betweenness and closeness are often unreliable [42, 43]. Therefore, this study utilised the strength centrality index in the analysis. The centrality indices were computed using the centrality plot function in the *R* package qgraph (Version 1.9.8) to assess the importance of each node in the network [28]. Additionally, this study utilised the bridge function of the *R* package networktools (Version 1.6.0) to calculate the bridge centrality index for bridge strength and to identify bridge symptoms. Within the network, bridge symptoms are those that act as crucial links between two groups of symptoms, facilitating the activation or escalation of symptoms across different areas clusters [28]. Identifying these bridge symptoms can reveal key targets for intervention, offering a new approach to developing precise management strategies.

#### 4.4.2. Network Stability and Accuracy

The stability and accuracy of the network were evaluated using the *R* package bootnet (Version 1.6) [40]. First, the accuracy of the edge weights​ is estimated using the nonparametric bootstrap method, with 95% confidence intervals (95% CIs). A narrower confidence interval indicates more precise edge estimation and greater confidence in the network [41]. Second, the stability of EIs and bridge edge weights​ was assessed using the case‐dropping bootstrap method and quantified by the correlation stability coefficient (CS‐C). CS‐C values above 0.5 indicate strong stability [41]. In this study, the bootstrap program was run 2000 times. Finally, bootstrap difference tests rely on 95% CIs to assess the disparity between two edges or the difference in strength between two nodes [41].

#### 4.4.3. Network Comparison

The study assessed network structural invariance, edge invariance and overall strength using the network comparison test (NCT) implemented in the *R* package NetworkComparisonTest (Version 2.2.2) [44]. In this study, global network strengths and structures were evaluated across subgroups (male vs. female; rural vs. urban), and the network structure was visualised accordingly.

#### 4.4.4. Bayesian Network Estimation

This study used a Bayesian network to analyse whether symptoms with high centrality were more likely to occur than other symptoms in the symptom network [31]. The methodology described in the relevant literature was used, and the analysis was conducted using the *R* package bnlearn (Version 5.0.2) analysis [45]. In this process, the hill‐climbing algorithm was applied, and the Bayesian information criterion (BIC) was used to learn the structure of the Bayesian network. Subsequently, 1000 iterations were performed to ensure a stable network structure. Each edge was considered valid if it appeared in at least 85% of the networks and its direction was consistently supported in more than 50% of them networks [46]. The thickness of an edge indicates the likelihood of its occurrence in the specified direction on the graph.

### 4.5. Ethical Considerations

The cross‐sectional study was approved by the Ethics Committee of Wenzhou Medical University, which approved the study design on 10 April 2024 (approval number 2024‐013). Written informed consent was obtained from all participants before they took part in the survey. All data were fully anonymised to ensure participants’ confidentiality and privacy, with all personal identifiers removed. The data were securely stored and accessible only to the research team.

## 5. Results

### 5.1. Descriptive Statistics

A total of 698 nursing interns were included in the network, with a mean age of 21.74 ± 1.14 years. Among them, 605 (86.68%) were female, and 356 (51.00%) were rural residents. The mean, SD, strength, skewness and kurtosis for all dimensions are presented in Table 1. The results showed that nursing interns in this study had mean disgust‐sensitivity and ageism scores that fell within the middle range of their respective scales. Regarding gerontological nursing career motivation, nursing interns showed moderate scores on the expectation dimension but lower scores on the value‐related dimensions, including interest, practicality, importance and cost.

**TABLE 1:** Descriptive statistics of the disgust sensitivity, ageism and gerontological nursing career motivation dimensions.

Dimension abbreviations	Dimension content	Mean (SD)	Strength	Skewness	Kurtosis
D1	Core disgust	27.60 (6.69)	0.89	0.05	−0.24
D2	Animal‐reminder disgust	15.98 (5.78)	0.72	0.23	0.01
D3	Contamination‐based disgust	9.34 (3.62)	0.79	0.32	−0.03
D4	Avoidance	16.04 (3.08)	0.90	−0.10	0.68
D5	Antilocution	15.27 (3.01)	1.06	−0.36	0.81
D6	Discrimination	17.56 (2.84)	0.64	−0.09	1.33
D7	Expectation	19.47 (4.09)	1.01	0.10	0.77
D8	Interest	10.06 (1.96)	1.00	−0.01	0.96
D9	Practicality	9.61 (2.20)	0.83	−0.01	0.90
D10	Importance	15.60 (3.63)	0.88	−0.01	0.84
D11	Cost	9.36 (2.27)	0.28	−0.05	0.67

*Note:* D1–D3, disgust sensitivity; D4–D6, ageism; D7, expectation; D8–D11, value.

Abbreviation: SD, standard deviation.

### 5.2. Network Structure

**FIGURE 1:**
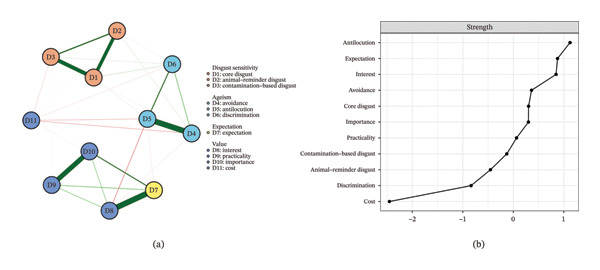
Disgust sensitivity, ageism and gerontological nursing career motivation among nursing interns. The (a) panel shows the visualisation of the network structure; the (b) panel shows the value of strength and expected influence in order.

**FIGURE 2:**
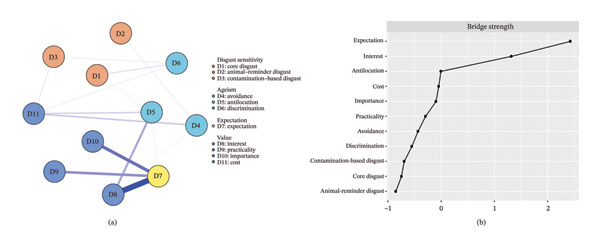
Network structure of disgust sensitivity, ageism and gerontological nursing career motivation showing bridge symptoms in nursing interns. The (a) panel shows the visualisation of the network structure of bridging symptoms; the (b) panel shows the value of bridge strength in order.

The network linking disgust sensitivity, ageism and gerontological nursing career motivation among nursing interns is shown in Figure 1, while the corresponding partial correlation matrix is provided in Supporting Table 1. The network consists of 11 nodes and 28 active edges. Among disgust sensitivities, the ‘core disgust’–‘contamination‐based disgust’ (D1‐D3) showed a strong connection, followed by the ‘core disgust’–‘animal‐reminder disgust’ (D1‐D2). Among ageism, the ‘avoidance’–‘antilocution’ (D4‐D5) showed the strongest connection. Among expectation and value, the ‘expectation’–‘interest’ (D7‐D8) showed a strong connection, followed by the ‘practicality’–‘importance’ (D9‐D10). Notably, ‘cost’ (D11) shows negative correlations with several nodes (D11‐D3/D4/D5/D6). Additionally, ‘expectation’ shows significant negative correlations with ‘antilocution’, ‘interest’ and ‘cost’ (D5‐D7/D8/D11). ‘Antilocution’ (D5) had the highest node strength across the entire network among nursing interns, followed by ‘expectation’ (D7). Among bridge symptoms, ‘expectation’ (D7) showed the highest bridge strength, followed by ‘interest’ (D8) (Figure 2).

### 5.3. Network Stability

**FIGURE 3:**
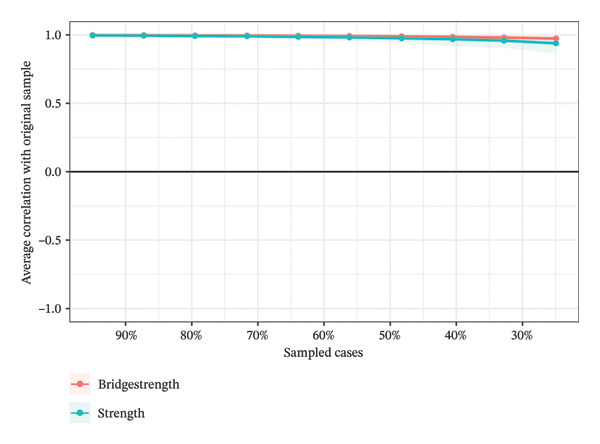
Using the case‐dropping bootstrap method, the stability of both the strength and bridge strength is examined. The *X*‐axis plotted the percentage of cases from the original sample included at each stage. The *Y*‐axis represented the average correlation between the centrality measures from the original network and the reestimated centrality measures from networks that progressively exclude a growing percentage of cases.

The case‐dropping bootstrap method showed that the symptom network developed in this research was considerably stable (CS‐C = 0.75), even when 75% of the samples were excluded (Figure 3). Furthermore, the nonparametric bootstrap method indicated that most comparisons of node strength were statistically significant (Supporting Figure 1). The EIs were also statistically significant (Supporting Figure 2), and the narrow bootstrapped 95% confidence intervals indicated that the edges were reliable (Supporting Figure 3).

### 5.4. Network Comparisons

There was no significant difference in overall network strength between male and female nursing interns (male: 4.220 vs. female: 4.566, *S* = 0.346, *p* = 0.376), in the distribution of EIs within the network (*M* = 0.200, *p* = 0.455) or in individual EIs (all *p* values > 0.05 after Holm–Bonferroni corrections) (Supporting Figure 4). Similarly, the strength of the global network did not differ significantly between nursing interns from urban and rural areas (urban: 4.562 vs. rural: 4.727, *S* = 0.165, *p* = 0.386), network structure distribution of EIs (*M* = 0.132, *p* = 0.752) or individual EIs (all *p* values > 0.05 after Holm–Bonferroni corrections) (Supporting Figure 5).

### 5.5. Bayesian Network Structure

**FIGURE 4:**
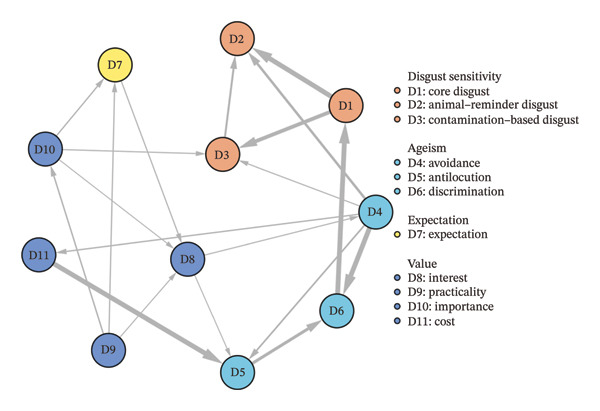
Bayesian network structure of disgust sensitivity, ageism and gerontological nursing career motivation dimensions of nursing interns. The arrows indicate the hypothesised direction of causality, and the thickness of the edges reflects the confidence in the predicted direction.

Figure 4 shows the DAG resulting from the Bayesian network analysis. A total of 26 directed edges were consistently present in 85% of the networks, and their direction was observed in more than 50% of cases. In this network, ‘avoidance’ (D4), ‘practicality’ (D9), ‘importance’ (D10) and ‘core disgust’ (D1) show strong probabilistic directional associations. ‘Avoidance’ (D4) shows the strongest probabilistic directional associations, linking it to multiple nodes. It is statistically associated with the ageism group (D4‐D6) and the disgust sensitivity group (D4‐D2/D3, D4‐D3‐D2). In addition, ‘avoidance’ (D4) is probabilistically connected to both the ageism group and the disgust‐sensitivity group through the value group (D4‐D11‐D5‐D6‐D1‐D2/D3), which represents the most likely pathway in the network. It is noteworthy that in this pathway, ‘cost’ (D11) plays a crucial role as a connecting node among the three dimensions. Following ‘avoidance’ (D4), ‘practicality’ (D9) and ‘importance’ (D10) also showed notable probabilistic directional associations, each linking to three dimensions and then connecting to other symptom groups. Within the disgust‐sensitivity group, ‘core disgust’ (D1) served as the central node, probabilistically linked to both ‘animal‐reminder disgust’ (D2) and ‘contamination‐based disgust’ (D3).

## 6. Discussion

To our knowledge, no prior research has examined the relationship between nursing interns’ disgust sensitivity, ageism and their motivation to pursue a career in gerontological nursing. This study uniquely uses a network analysis approach to explore the complex correlations and interrelationships among them and to identify the key nodes involved. By identifying these critical nodes, interventions can be developed more effectively, thereby increasing the effectiveness of the measures. The findings offer new insights into the psychological mechanisms underlying nursing interns’ career choices in geriatric nursing and have significant implications for developing effective intervention strategies.

### 6.1. The Characteristics of Disgust Sensitivity, Ageism and Gerontological Nursing Career Motivation Among Nursing Interns

The descriptive statistics showed that the nursing interns in this study generally exhibited moderate levels of aversion sensitivity and ageism, which aligns with findings from previous studies and indicates that these factors are common concerns [47, 48]. This finding highlights the persistence of these attitudes among nursing interns, despite ongoing efforts to address them within nursing education and training.

Although interns may expect a career in geriatric care, many do not view it as highly rewarding or prestigious, which can affect their career decisions [49]. To increase motivation, nursing programmes could incorporate modules that emphasise the societal and personal importance of geriatric nursing. This could include guest lectures from experienced geriatric nurses, mentorship programmes and internships with early exposure. Additionally, targeted training to reduce disgust sensitivity and ageism could include cognitive‐behavioural therapy, role‐play, case discussions, structured, supervised interactions with older patients and reflective exercises.

Nursing education reforms should address geriatric career motivation by integrating value‐focused content and early exposure to ageing and by implementing targeted interventions to reduce disgust sensitivity and ageism.

### 6.2. Identification and Practical Significance of Network Structure and Key Nodes

Nodal analysis revealed that ‘antilocution’ (D5) had the highest strength in the overall network and that there was a significant negative correlation with gerontological nursing career motivation (D5‐D7/D8/D11). This suggests that antilocution in ageism may be a prominent expression of nursing interns’ negative attitudes toward older adults and that these attitudes are also associated with their motivation to pursue careers in geriatric nursing. The study by Pang [50] has shown that age discrimination against nursing interns influences their motivation to pursue a career in geriatric nursing. Identifying strong nodes, such as ‘antilocution’ (D5), can thus offer specific intervention points for educational and clinical programs. For example, antiageism training could be included in clinical internships through guided reflection sessions, intergenerational communication workshops and supervised interactions with older patients, helping interns critically examine stereotypes and improve their attitudes toward older adults.

In the future, ageing education should be integrated with clinical practice to reduce age discrimination against older adults by nursing interns and potentially influence their motivation to pursue geriatric nursing careers [51, 52].

Among disgust sensitivities, ‘core disgust’ (D1) was at the centre and showed strong correlations with both ‘animal‐reminder disgust’ (D2) and ‘contamination‐based disgust’ (D3). Disgust sensitivity might be linked to care‐avoidant behaviours when interacting with the elderly patients [16]. However, research shows that higher levels of empathy and morality can reduce personal aversion and enhance the quality of care [53, 54]. Future interventions could involve gradual exposure during clinical placements, such as stepwise participation in tasks related to elderly care, under instructor supervision. These could be combined with empathy‐focused reflection exercises to reduce the connection between D1 and D2/D3 and enhance the acceptance of geriatric care. The effectiveness of these strategies could be assessed by measuring disgust sensitivity and attitudes toward older adults before and after training.

‘Expectation’ (D7) functioned as a bridging node, positively correlating with career ‘interest’ (D8), ‘practicality’ (D9) and “importance” (D10). This suggests that enhancing nursing interns’ expectations of geriatric care could be associated with improved professional attitudes. Previous studies have shown that ageism lowers expectations for gerontological nursing interns’ careers [22]. Our results also found that ‘antilocution’ (D5), a central node of ageism, is significantly negatively correlated with a gerontological nursing career (D7/D8/D11). Combined with previous studies [22], this further confirms that ageism may reduce nursing interns’ motivation to choose a gerontological nursing career. Additionally, the links between ‘expectation’ (D7) and factors such as ‘interest’ (D8), ‘practicality’ (D9) and ‘importance’ (D10) suggest that the limited perceived value and relevance of geriatric nursing may also reduce nursing interns’ motivation to pursue this career path [50]. Educational institutions and clinical training sites can address this by providing structured geriatric career guidance during internships. This might include mentorship from experienced geriatric nurses, case‐based discussions of successful geriatric care and opportunities to observe advanced geriatric practices.

The negative correlation between ‘cost’ (D11) and both disgust sensitivity and ageism shows that nursing interns with higher disgust sensitivity and more ageist attitudes tend to perceive costs as lower. This indicates that nursing interns devote less time and emotional effort to geriatric care, as shown by Dai et al. al [3]. It can be seen that those who are less sensitive to disgust and hold fewer ageist attitudes may be more aware of, or more concerned about, the emotional and time‐related demands of the profession. Therefore, practical interventions in clinical settings could reduce perceived emotional costs by providing supportive supervision, regular mentor feedback and team‐based care models that better manage workload and emotional demands. Additionally, regular evaluations of interns’ workloads and emotional stress could help institutions modify training programmes to further improve motivation [55, 56].

### 6.3. Network Stability and Cross‐Group Coherence

The network in this study is stable, with consistent, reproducible relationships among dimensions that can inform future interventions and enhance their effectiveness. The network showed high stability (CS‐C = 0.75), indicating reliable results and supporting the development of targeted interventions.

Additionally, network comparisons among nursing interns of different genders and residences were not statistically significant, suggesting that the patterns of correlation among disgust sensitivity, ageism and gerontological nursing career motivation are likely consistent across these demographic groups. This suggests that these psychological and motivational factors operate similarly across demographic groups, with significant implications for the design and implementation of intervention programmes.

Therefore, training programmes aimed at improving motivation in geriatric nursing may adopt a unified framework rather than designing separate programmes for different demographic groups. Resources can thus be allocated more efficiently, enabling the development of universal strategies to target the key factors influencing nursing interns’ career motivation in geriatric care. For example, standardised educational modules, shared clinical training activities and common evaluation tools could be implemented across nursing schools and internship programs. This strategy may help ensure consistent training quality and enhance the efficiency of intervention delivery.

### 6.4. Probabilistic Directional Paths and Dynamic Interventions in Bayesian Networks

‘Avoidance’ (D4), a central node of the network, is probabilistically associated with value (D11), ageism (D4‐D5‐D6) and disgust sensitivity (D4‐D2/D3), forming a tightly connected network of factors (D4‐D11‐D5‐D6‐D1‐D2/D3). These associations highlight patterns that may be linked to negative attitudes and reduced willingness among nursing interns to pursue a career in geriatric nursing. Early‐stage interventions targeting ‘avoidance tendencies’, such as simulated exposure to situations, could influence these patterns, fostering more positive perceptions and reducing resistance toward geriatric care [57].

Additionally, the complex links between ‘practicality’ (D9) and ‘importance’ (D10) suggest that emphasising the professional value of geriatric nursing in curriculum design may be indirectly associated with lower disgust sensitivity and ageism. It is worth noting that ‘cost’ (D11), as an essential intermediary node, underscores the importance of improving professional treatment and working conditions in the elderly care sector industry [58, 59]. Practical measures such as adjusting workloads, offering mentorship and establishing clear career paths could reduce perceived barriers and make geriatric nursing more appealing [60].

Furthermore, the Bayesian network results of this study indicate that nursing interns’ expectations of a career in gerontological nursing may be probabilistically linked to ageism, contradicting previous research findings [22, 61]. These findings emphasise the importance of multifactor assessment when developing interventions, incorporating probabilistic relationships to tailor strategies to various intern profiles. The approach provides a more accurate and adaptable framework for future research and practice.

## 7. Limitations

This study has some limitations. Firstly, the cross‐sectional design limits causal inference. Although Bayesian networks and network analysis can identify probabilistic directional relationships and important connections between variables, they cannot establish causality. Future studies using longitudinal or experimental designs, such as randomised controlled trials, are necessary to better explore potential causal pathways between them.

Secondly, relying on self‐report measures may introduce subjectivity and recall bias. Future research would benefit from incorporating objective indicators, such as behavioural observations and physiological responses, to provide a more thorough and accurate evaluation.

Thirdly, the study’s gender distribution was heavily skewed. This imbalance substantially reduces the statistical power of the NCT, increasing the risk of Type II errors, in which meaningful differences in global network strength or EIs go undetected. Future research should prioritise recruitment strategies that ensure more balanced gender representation to enhance the robustness and generalisability of the comparative results.

## 8. Conclusion

In this study, the complex interactions among nursing interns’ disgust sensitivity, ageism and gerontological nursing career motivation were revealed for the first time using network modelling. It was found that ‘antilocution’ (D5) in ageism and ‘expectation’ (D7) in career motivation function as core nodes, showing strong associations with negative attitudes and lower career identity. Additionally, ‘avoidance’ (D4) is probabilistically associated with higher disgust sensitivity (D4‐D3/D2) and higher ageism (D4‐D5‐D6), indicating a robustly interconnected network of psychological factors. Despite interns’ moderate career aspirations in geriatric nursing, low identification with career values (D9/D10) and high perceived costs (D11) constitute a major barrier. The network’s strong stability (CS‐C = 0.75) and consistency across demographic groups suggest that intervention strategies could target early efforts to counteract avoidance tendencies, increase perceived career value and improve the work environment to reduce emotional costs. In the future, we should validate causal pathways through longitudinal studies and broaden the study’s applicability by including cross‐cultural samples, thereby providing empirical evidence for developing a precise intervention system targeting network mechanisms.

## Author Contributions

Yiqian Tang: conceptualisation, data curation, formal analysis, methodology, software, visualisation, writing–original draft and writing–review and editing. Chenxi Zhu: data curation, investigation and software. Liling Xu: investigation. Lilu Wang: investigation and data curation. Yawen Hu: investigation. Lianlian Zhu: conceptualisation, methodology, data curation, writing–review and editing and project administration. Yechun Gu: conceptualisation, methodology, data curation and project administration. Hongbo Xu: conceptualisation, methodology, data curation, writing–review and editing and project administration.

## Funding

No funding was received for this manuscript.

## Conflicts of Interest

The authors declare no conflicts of interest.

## Supporting Information

Additional supporting information can be found online in the Supporting Information section.

## Supporting information


**Supporting Information** The Supporting information includes one table and five figures. Supporting Table 1 shows the correlation matrix. Supporting Figure 1 and Figure 2 show the bootstrapped stability test. Supporting Figure 3 shows the bootstrapped confidence intervals. Supporting Figure 4 and Figure 5 show the network comparison of gender and residence.

## Data Availability

The data used to support the findings of this study are available from the corresponding author upon request.
