# Supplementary material for: Nursing Interns’ Disgust Sensitivity, Ageism and Gerontological Nursing Career Motivation: A Network Analysis
Source: J Nurs Manag. 2026 May 18;2026:9944227. doi: 10.1155/jonm/9944227 (PMC13181326; doi:10.1155/jonm/9944227)
Supplement: Supplementary file 1 — Supporting Information The Supporting information includes one table and five figures. Supporting Table 1 shows the correlation matrix. Supporting Figure 1 and Figure 2 show the bootstrapped stability test. Supporting Figure 3 shows the bootstrapped confidence intervals. Supporting Figure 4 and Figure 5 show the network comparison of gender and residence. [file JONM-2026-9944227-s001.docx]

Supplementary materials

Supplementary Table 1 Correlation matrix of the disgust sensitivity, ageism and gerontological nursing career motivation dimensions

Supplementary Figure 1 Bootstrapped stability test for node-weight

Supplementary Figure 2 Bootstrapped stability test for edge-weight

Supplementary Figure 3 Bootstrapped confidence intervals of edge weights

Supplementary Figure 4 Network comparisons of the disgust sensitivity, ageism and gerontological nursing career motivation dimensions by gender in nursing interns

Supplementary Figure 5 Network comparisons of the disgust sensitivity, ageism and gerontological nursing career motivation dimensions by urban and rural nursing interns

Supplementary Table 1 Correlation matrix of the disgust sensitivity, ageism and gerontological nursing career motivation dimensions

|  | D1 | D2 | D3 | D4 | D5 | D6 | D7 | D8 | D9 | D10 | D11 |
| --- | --- | --- | --- | --- | --- | --- | --- | --- | --- | --- | --- |
| D1 | 1.00 |  |  |  |  |  |  |  |  |  |  |
| D2 | 0.64 | 1.00 |  |  |  |  |  |  |  |  |  |
| D3 | 0.66 | 0.59 | 1.00 |  |  |  |  |  |  |  |  |
| D4 | 0.13 | 0.19 | 0.19 | 1.00 |  |  |  |  |  |  |  |
| D5 | 0.12 | 0.17 | 0.16 | 0.71 | 1.00 |  |  |  |  |  |  |
| D6 | 0.24 | 0.20 | 0.22 | 0.55 | 0.57 | 1.00 |  |  |  |  |  |
| D7 | -0.09 | -0.08 | -0.03 | -0.27 | -0.40 | -0.19 | 1.00 |  |  |  |  |
| D8 | -0.11 | -0.09 | -0.03 | -0.35 | -0.47 | -0.22 | 0.84 | 1.00 |  |  |  |
| D9 | -0.07 | -0.04 | 0.01 | -0.20 | -0.29 | -0.14 | 0.76 | 0.73 | 1.00 |  |  |
| D10 | -0.09 | -0.03 | 0.01 | -0.21 | -0.31 | -0.17 | 0.78 | 0.74 | 0.81 | 1.00 |  |
| D11 | -0.10 | -0.13 | -0.15 | -0.30 | -0.29 | -0.23 | 0.03 | 0.06 | 0.07 | 0.02 | 1.00 |

The results of the bootstrapped difference tests (α=0.05) for node weights are shown in this figure. There was a total of 11 dimensions. The color of the boxes indicates whether node weights differ significantly from each other (i.e., black) or do not differ significantly (i.e., grey). The diagonal line indicates the strength of node-weights.


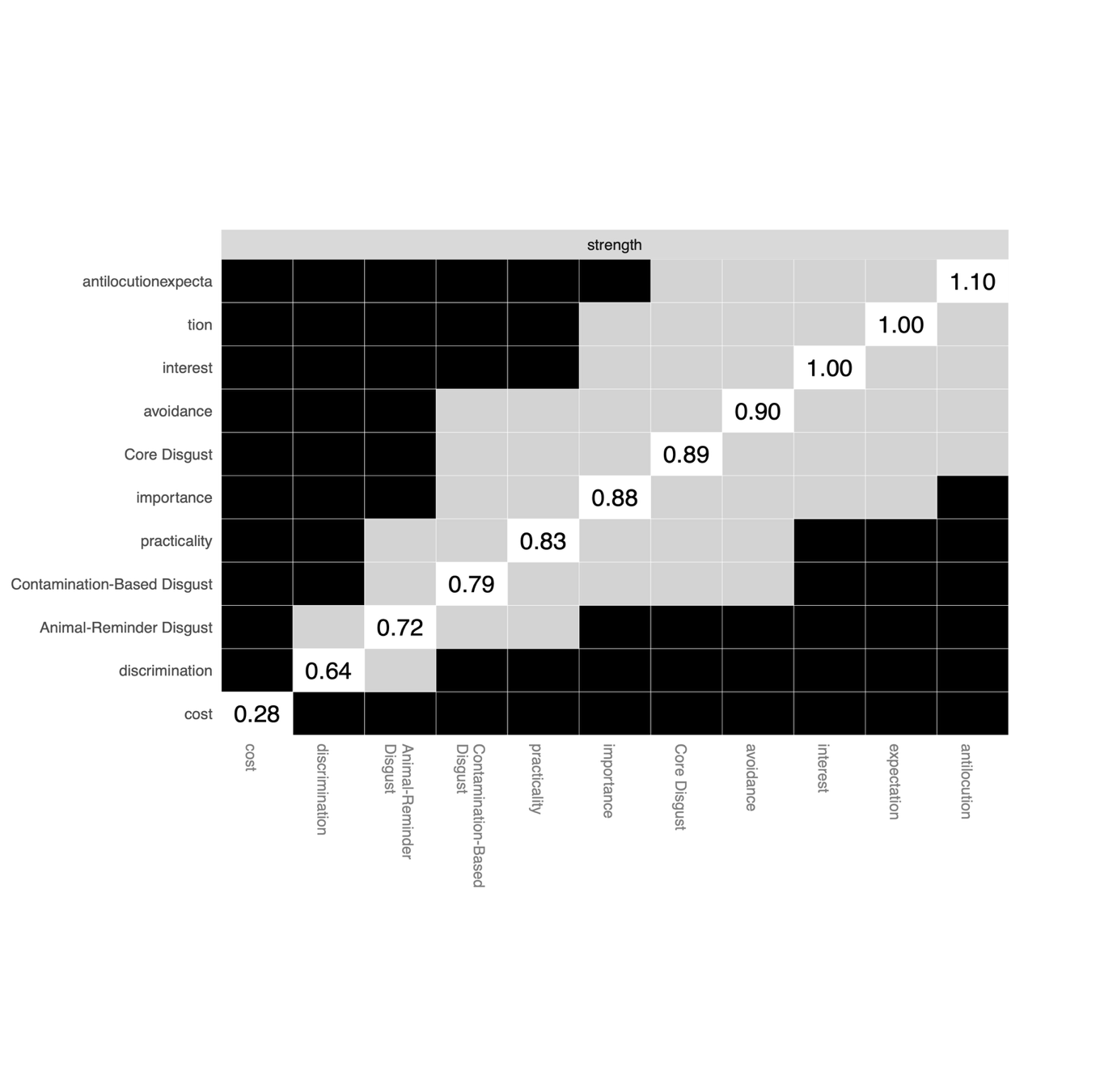


Supplementary Figure 1 Bootstrapped stability test for node-weight

The results of the bootstrapped difference tests (α=0.05) for edge-weights are shown in this figure. There was a total of 28 edges between all 11 dimensions. The color of the boxes indicates whether edge weights differ significantly from each other (i.e., black) or do not differ significantly (i.e., grey). The diagonal line indicates the strength of edge-weights.


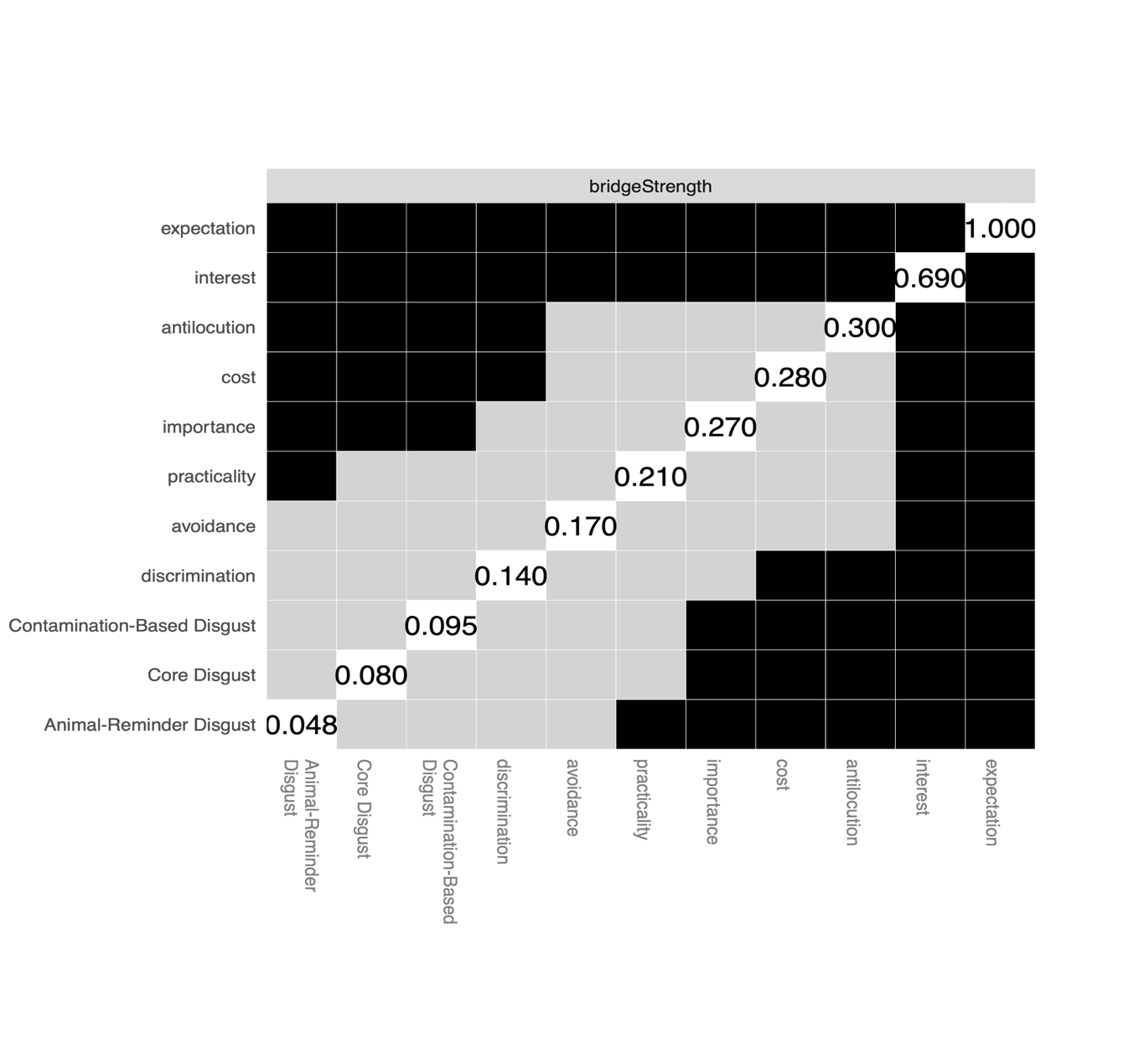


Supplementary Figure 2 Bootstrapped stability test for edge-weight

The red dots represent the values of each edge weight directly from the network, while the black dots signify the bootstrap estimates of these edge weights, arranged in descending order of value. The grey shaded area denotes the 95% Confidence Intervals for the edge weights, which were calculated using the non-parametric bootstrap method. A wider interval suggests less stability, whereas a narrower interval suggests greater stability.


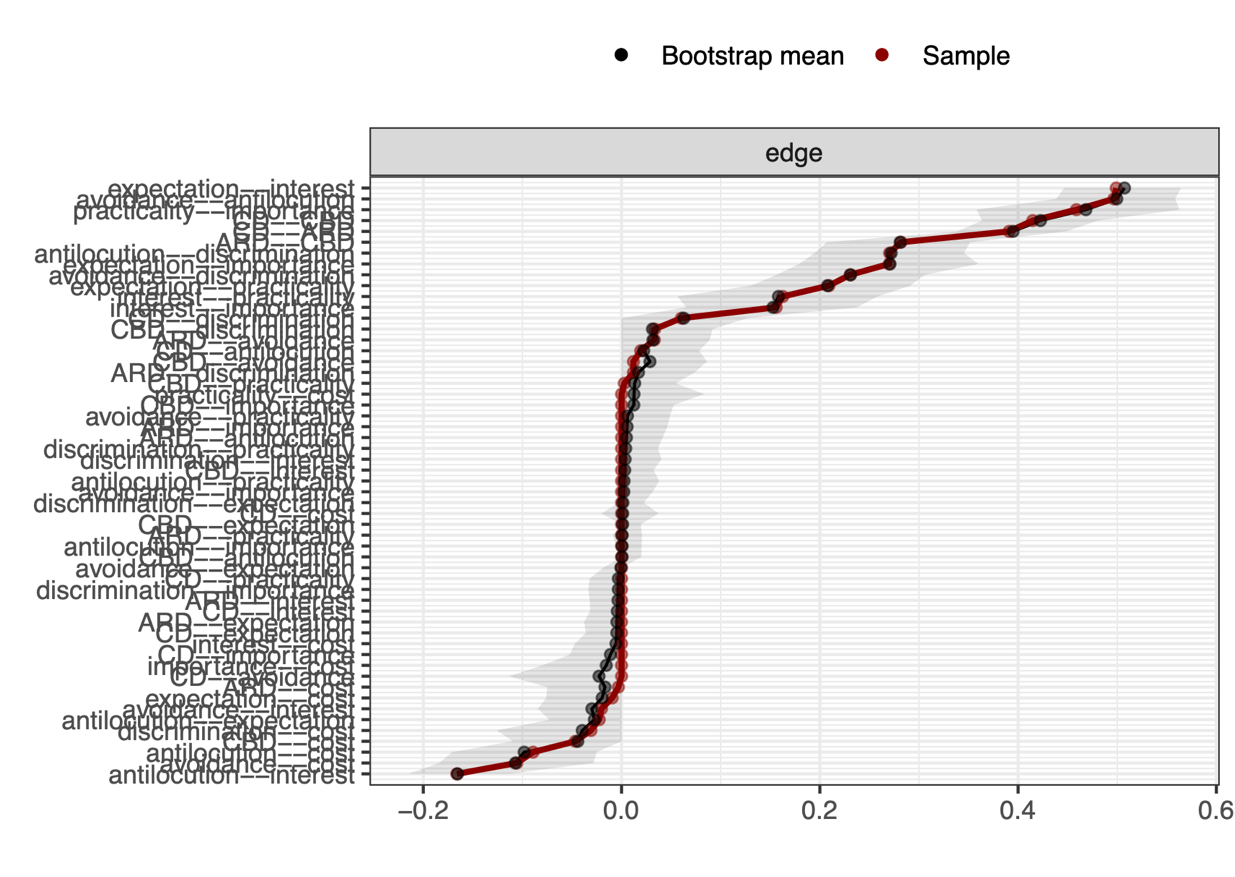


Supplementary Figure 3 Bootstrapped confidence intervals of edge weights


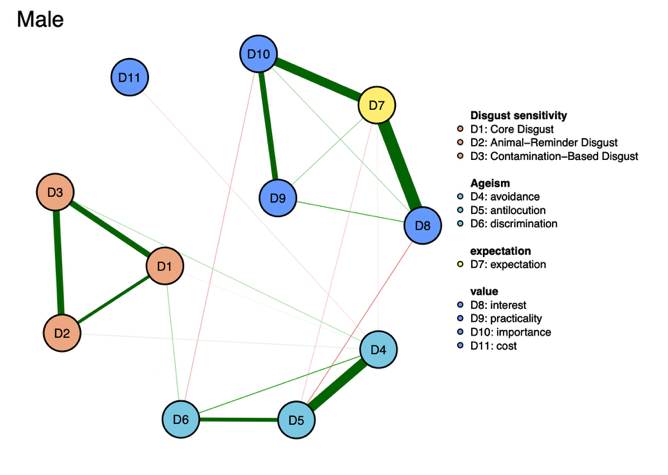

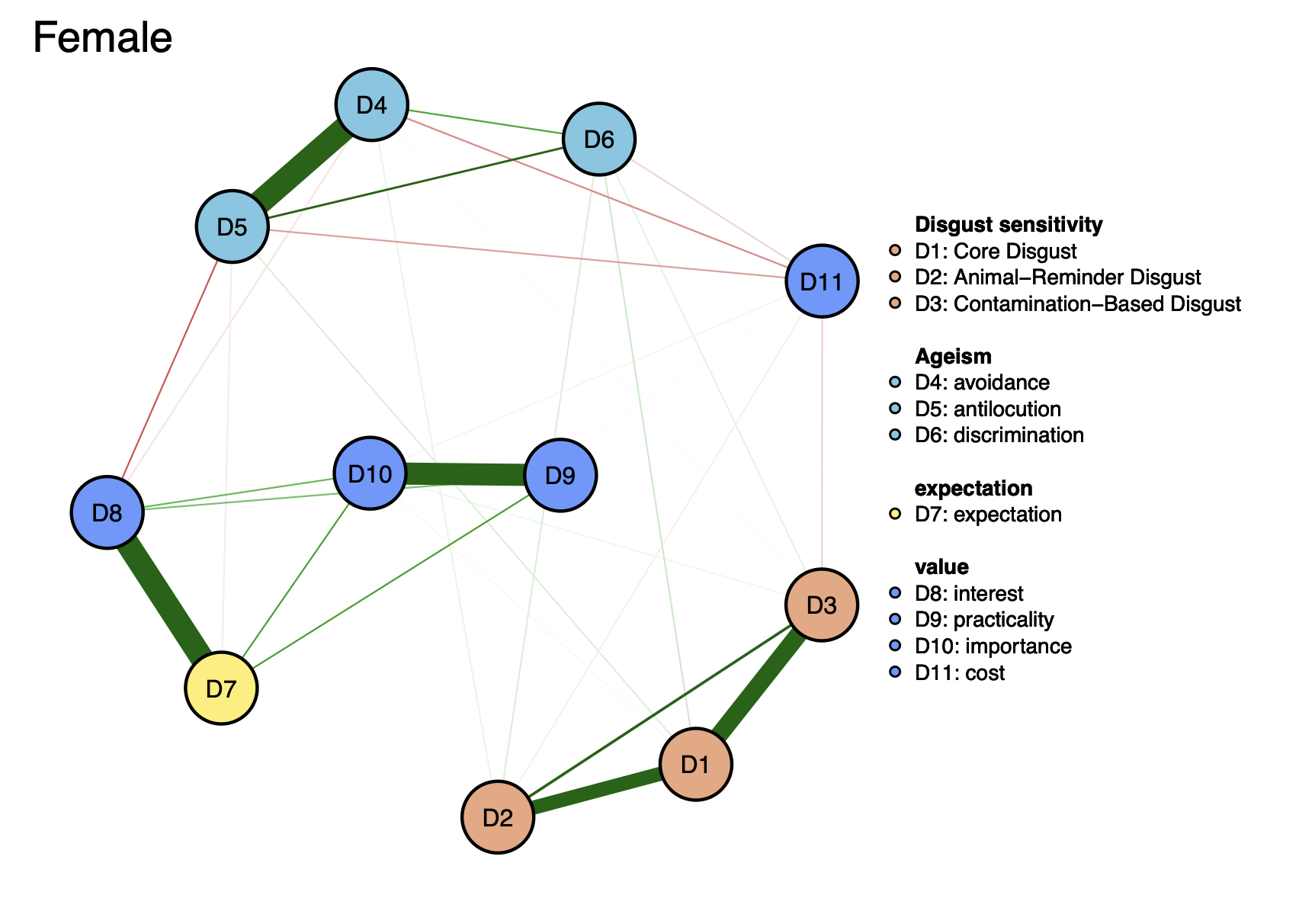


Supplementary Figure 4 Network comparisons of the disgust sensitivity, ageism and gerontological nursing career motivation dimensions by gender in nursing interns


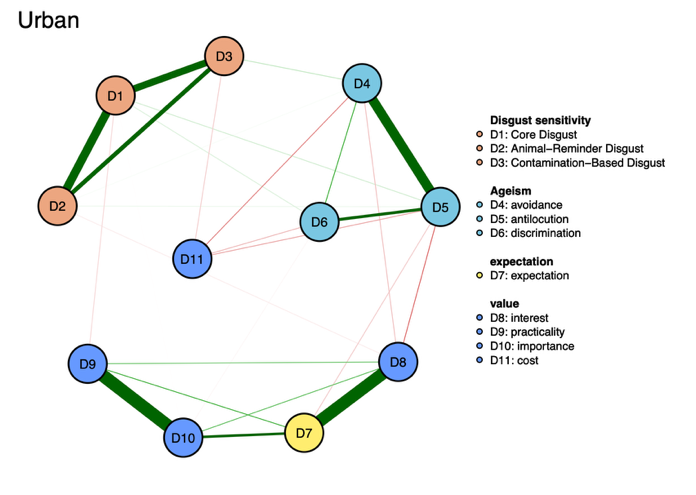

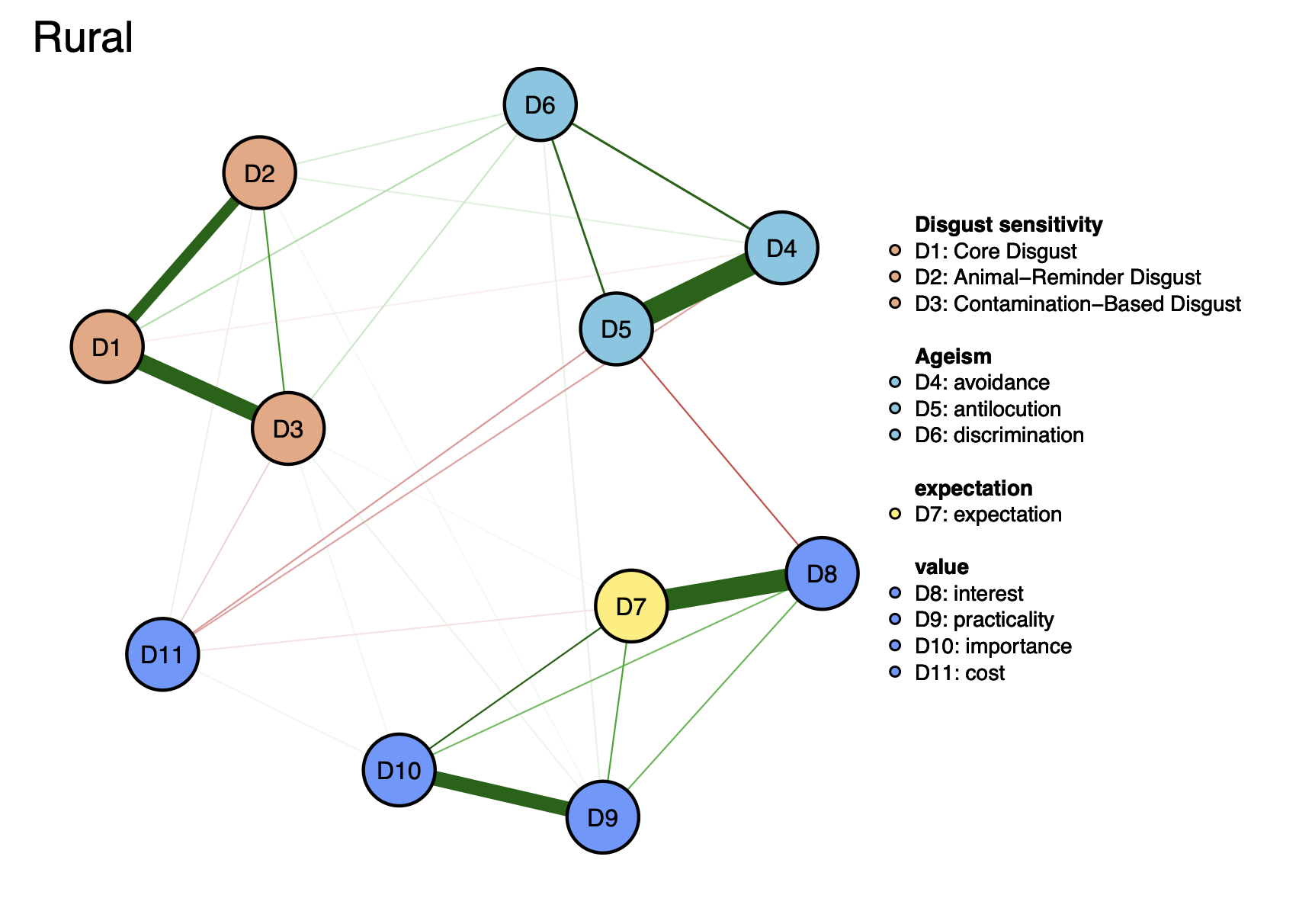


Supplementary Figure 5 Network comparisons of the disgust sensitivity, ageism and gerontological nursing career motivation dimensions by urban and rural nursing interns
